# Pseudo-Tuberculosis

**Published:** 1899-05-27

**Authors:** 


### PSEUDO-TUBERCULOSIS.

If the recommendations of the committee appointed by the Pathological Society to consider the nomenclature of the conditions sometimes described as pseudo-tuberculosis should meet with general acceptation, much confusion of language and much resulting scientific inaccuracy will be done away with. The term "pseudo-tuberculosis" has been applied to several distinct morbid processes which agree with one another only in the fact of producing in the tissues small nodular masses which have been considered to resemble "tubercles." These lumpy or tubercular conditions may arise from a great variety of infections—in fact, one may almost say that any infection which tends to be implanted at many points, and to grow locally wherever it is sown, tends to develop lumps or tubercles; and thus, from a mere misuse of words, a whole host of pseudo-tuberculoses have come into existence. The committee say that they think that confusion has arisen from the employment of the word "tubercle" in two senses: (1) As a general anatomical term for a small nodule; (2) in a specific sense for the nodular lesions of the disease produced by the tubercle bacillus of Koch; and they recommend that the word should no longer be used as a general anatomical term, but that, if used at all, it should be only as a designation of the nodular lesions produced by the tubercle bacillus. To prevent ambiguity, however, they suggest that all lesions having the form of "tubercles" should be called generally "nodules," those produced by Koch's bacillus being distinguished as "tuberculous nodules"; and that the nodules produced by other causes should be distinguished by a prefix indicative of their cause, if known; as, for example, "glanders nodule," "aspergillar nodule"; or, if their cause is not known, by some distinctive designation not involving any reference to the word "tubercle."

